# Supplementary material for: Circulating Immune Proteins: Improving the Diagnosis and Clinical Outcome in Advanced Non-Small Cell Lung Cancer
Source: Int J Mol Sci. 2023 Dec 18;24(24):17587. doi: 10.3390/ijms242417587 (PMC10743468; doi:10.3390/ijms242417587)
Supplement: Supplementary file 1 [file ijms-24-17587-s001.zip › ijms-2656693-Supplementary Figures.pdf]

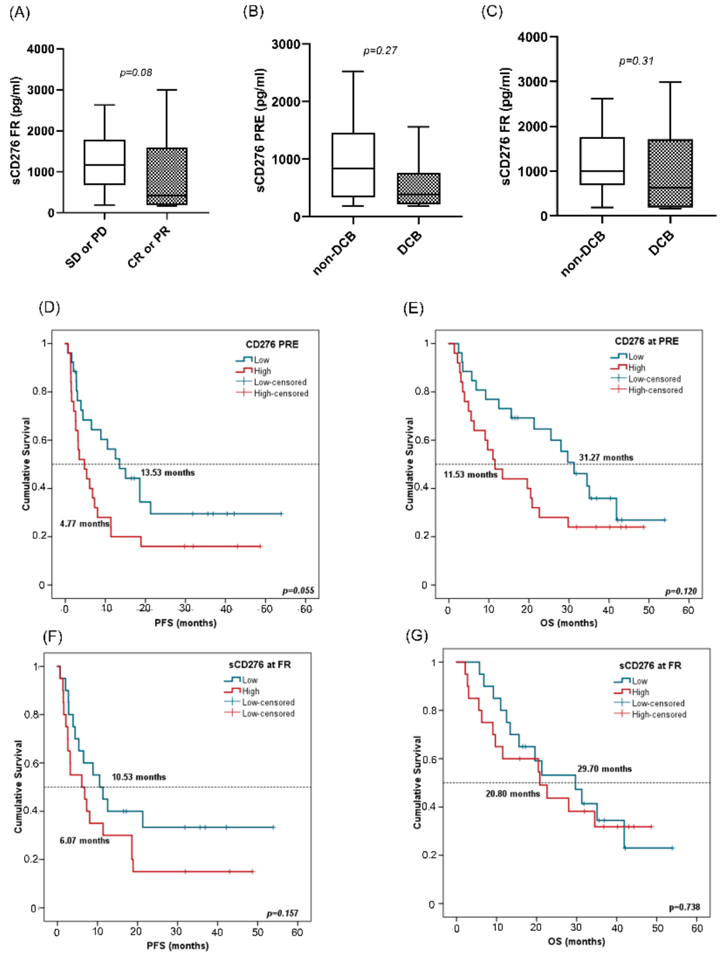

Figure S1. sCD276 and tumor response, clinical benefit and survival. (A) sCD276 levels at first response assessment in patients who were non-responders (n=24) compared to responders to pembrolizumab (n=16). (B) sCD276 levels at baseline in patients without durable clinical benefit (n=23) and patients with durable clinical benefit (n=29). (C) sCD276 levels at first response assessment in patients without durable clinical benefit (n=16) and patients with durable clinical benefit (n=24). (D-E) Progression-free survival (PFS) and Overall survival (OS) according to sCD276 levels at pretreatment. Cutoff values correspond to the median soluble levels. Red lines represent patients with high levels of sCD276, whereas blue lines represent patients with low levels of sCD276. (F-G) Progression-free survival (PFS) and Overall survival (OS) according to sCD276 levels at first response assessment. Cutoff values correspond to the median soluble levels. Red lines represent patients with high levels of sCD276, whereas blue lines represent patients with low levels of sCD276. P-values were calculated by Mann-Whitney test (A and C) or log-rank test (D-E). *SD*, stable disease; *PD*, progression; *PR*, partial response; *CR*, complete response; *PFS*, progression-free survival; *OS*, overall survival; *FR,* first response assessment; *PRE*, baseline; *DCB*, durable clinical benefit.

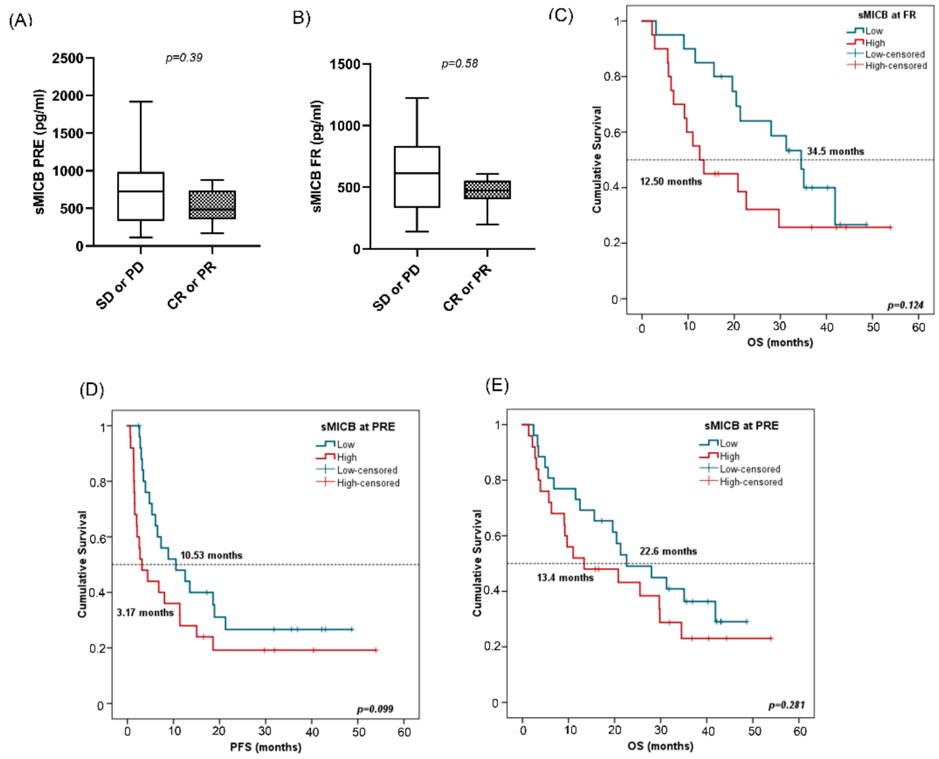

Figure S2. sMICB and tumor response and survival. (A) sMICB levels at baseline in patients who were non-responders (n=31) compared to responders to pembrolizumab (n=21). (B) sMICB levels at first tumor assessment (FR) in patients who were non-responders to pembrolizumab (n=24) compared to non-responders (n=16). (C) Overall survival (OS) according to sMICB levels at first tumor assessment. Cutoff values correspond to the median soluble levels. Red lines represent patients with high levels of sMICB, whereas blue lines represent patients with low levels of sMICB. (D) Progression-free survival (PFS) according to sMICB levels at baseline. Cutoff values correspond to the median soluble levels. Red lines represent patients with high levels of sMICB, whereas blue lines represent patients with low levels of sMICB. (E) Overall survival (OS) according to sMICB levels at baseline. Cutoff values correspond to the median soluble levels. Red lines represent patients with high levels of sMICB, whereas blue lines represent patients with low levels of sMICB. P-values were calculated by Mann-Whitney test (A and B) or log-rank test (D-H). *SD*, stable disease; *PD*, progression; *PR*, partial response; *CR*, complete response; *PFS*, progression-free survival; *OS*, overall survival; *FR,* first response assessment; *PRE*, baseline.

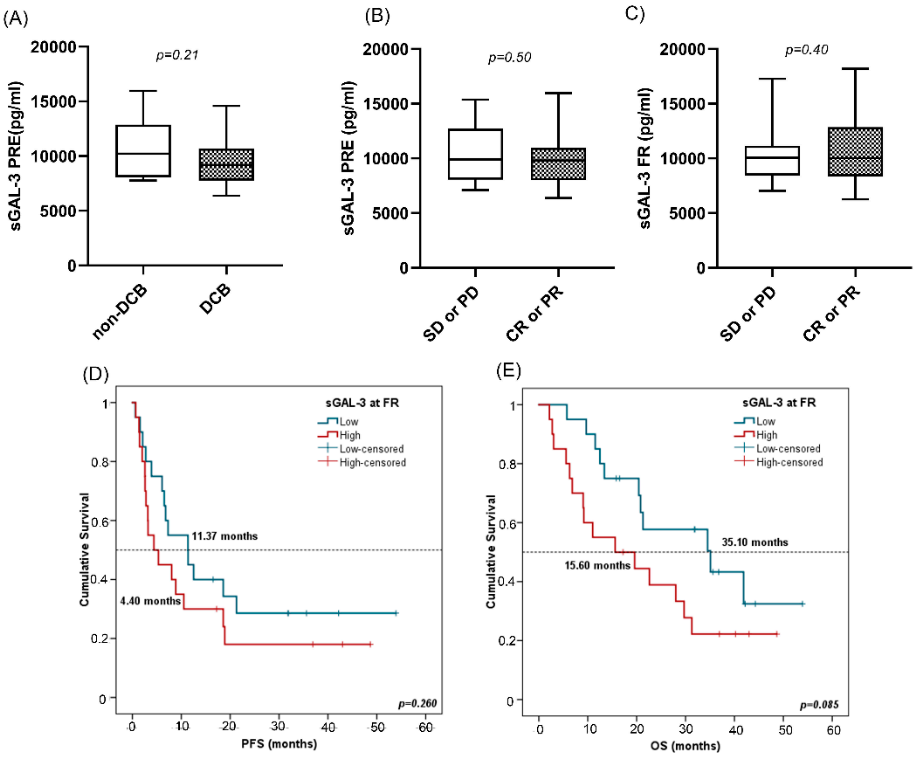

Figure S3. sGAL-3 and clinical benefit, tumor response and survival. A) sGAL-3 levels at baseline in patients without durable clinical benefit (n=23) and patients with durable clinical benefit (n=29). B) sGAL-3 levels at pretreatment in patients who were non-responders (n=31) compared to responders to pembrolizumab (n=21). C) sGAL-3 levels at first response assessment in patients who were non-responders (n=24) compared to responders to pembrolizumab (n=16). D-E) Progression-free survival (PFS) and Overall survival (OS) according to sGAL-3 levels at first response assessment. Cutoff values correspond to the median soluble levels. Red lines represent patients with high levels of sGAL-3, whereas blue lines represent patients with low levels of sGAL-3. P-values were calculated by Mann-Whitney test (A and C) or log-rank test (D-E). *SD*, stable disease; *PD*, progression; *PR*, partial response; *CR*, complete response; *PFS*, progression-free survival; *OS*, overall survival; *FR,* first response assessment; *PRE*, baseline; *DCB*, durable clinical benefit.

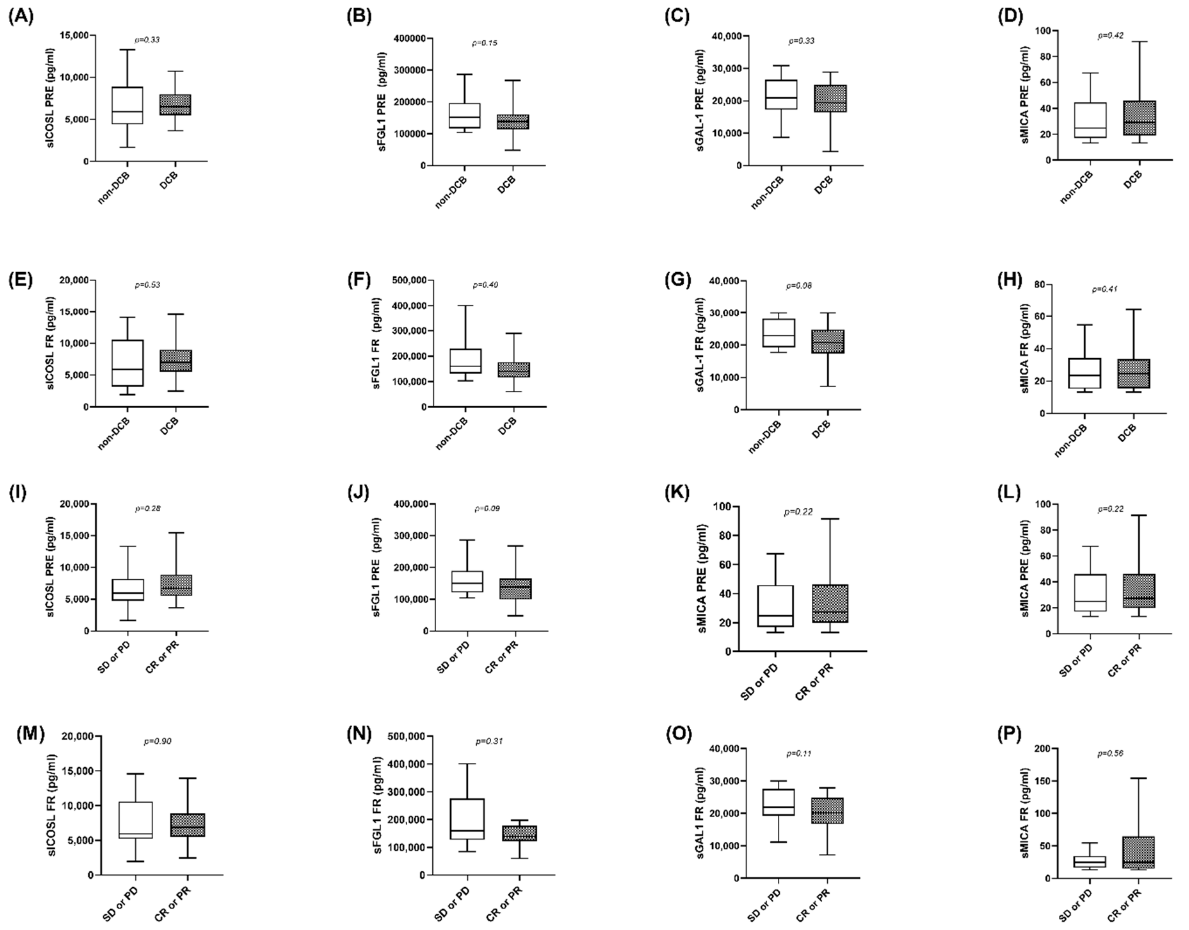

Figure S4. sICOSL, sFGL1, sGAL-1 and sMICA and tumor response and clinical benefit. (A-D) Biomarkers levels at baseline in patients without durable clinical benefit (n=23) and patients with durable clinical benefit (n=29). (E-H). Biomarkers levels at first response assessment in patients without durable clinical benefit (n=16) and patients with durable clinical benefit (n=24). (I-L) Biomarkers levels at baseline in patients who were non-responders (n=31) compared to responders to pembrolizumab (n=21). (M-P). Biomarkers levels at first response assessment in patients who were non-responders (n=24) compared to responders to pembrolizumab (n=16). P-values were calculated by Mann-Whitney test. *SD*, stable disease; *PD*, progression; *PR*, partial response; *CR*, complete response; *FR,* first response assessment; *PRE*, baseline; *DCB*, durable clinical benefit.

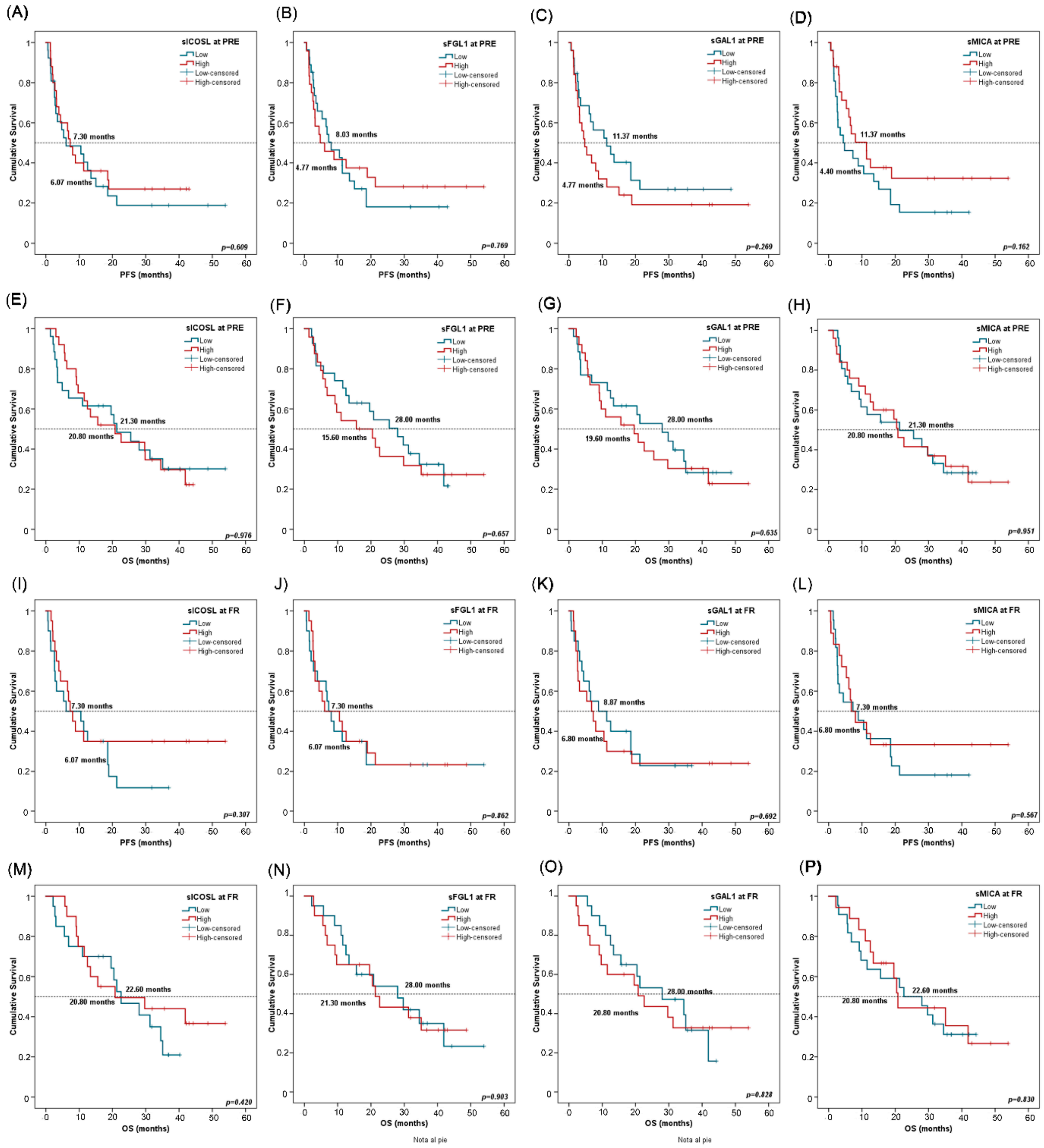

Figure S5. SICOSL, sFGL1, sGAL-1 and sMICA and survival. (A-D) Progression-free survival (PFS) according to biomarker levels at baseline. (E-H). Overall survival (OS) according to biomarker levels at baseline. (I-L) Progression-free survival (PFS) according to biomarker levels at first response assessment. (M-P). Overall survival (OS) according to biomarker levels at first response assessment. Cutoff values correspond to the median soluble levels. Red lines represent patients with high levels of sGAL-3, whereas blue lines represent patients with low levels of sGAL-3. P-values were calculated by log-rank test. *PFS*, progression-free survival; *OS*, overall survival; *FR,* first response assessment; *PRE*, baseline.
